# A systematic review of interventions to recognise, refer and diagnose patients with lung cancer symptoms

**DOI:** 10.1038/s41533-022-00312-9

**Published:** 2022-10-18

**Authors:** Mohamad M. Saab, Megan McCarthy, Michelle O’Driscoll, Laura J. Sahm, Patricia Leahy-Warren, Brendan Noonan, Serena FitzGerald, Maria O’Malley, Noreen Lyons, Heather E. Burns, Una Kennedy, Áine Lyng, Josephine Hegarty

**Affiliations:** 1 Catherine McCauley School of Nursing and Midwifery, University College Cork, Cork, Ireland; 2 School of Pharmacy, University College Cork, Cork, Ireland; 3 Rapid Access Lung Clinic, Cork University Hospital, Cork, Ireland; 4 National Cancer Control Programme, Health Services Executive, Dublin, Ireland

**Keywords:** Respiratory tract diseases, Diagnosis, Respiratory signs and symptoms

## Abstract

Patients with lung cancer (LC) often experience delay between symptom onset and treatment. Primary healthcare professionals (HCPs) can help facilitate early diagnosis of LC through recognising early signs and symptoms and making appropriate referrals. This systematic review describes the effect of interventions aimed at helping HCPs recognise and refer individuals with symptoms suggestive of LC. Seven studies were synthesised narratively. Outcomes were categorised into: Diagnostic intervals; referral and diagnosis patterns; stage distribution at diagnosis; and time interval from diagnosis to treatment. Rapid access pathways and continuing medical education for general practitioners can help reduce LC diagnostic and treatment delay. Awareness campaigns and HCP education can help inform primary HCPs about referral pathways. However, campaigns did not significantly impact LC referral rates or reduce diagnostic intervals. Disease outcomes, such as LC stage at diagnosis, recurrence, and survival were seldom measured. Review findings highlight the need for longitudinal, powered, and controlled studies.

## Introduction

Lung cancer (LC) is the most common cause of cancer incidence and mortality worldwide, with 2.1 million new cases and 1.8 million deaths in 2018^1^. It is estimated that, by 2040, the number of annual LC diagnoses and deaths will increase to 3.63 and 3.01 million respectively^2^. Worldwide, more than half of LCs (53%) are diagnosed in people aged between 55 and 74 years^3^. Data from 185 countries indicate that LC is typically diagnosed at an advanced stage, with a 5-year survival rate of 10–20%^4^.

LC has a relatively broad symptom signature compared to other cancers, such as breast and testicular cancers that typically present with a single identifiable symptom (e.g., painless lump)^5–7^. Early-stage LC can be asymptomatic or can cause a range of symptoms including a persistent cough, changes to an existing cough, shortness of breath, and chest pain^8,9^. Systemic symptoms, such as unexplained weight loss and fatigue, are typically associated with advanced disease^10^. Haemoptysis is one of the strongest symptom predictors of LC^8,11^. The broad symptom signature of LC, and overlap with common symptoms of benign disease, may contribute to delays in presentation and diagnosis^12^.

Early medical help-seeking for symptoms suggestive of LC is a key enabler of early diagnosis, curative treatment, and improved survival^11^. However, a Swedish study found that patients diagnosed with LC experience, on average, a 6-month delay between symptom onset and initiation of treatment^13^. Reasons for delayed patient help-seeking include patient factors, such as symptom misappraisal, fear of a potential cancer diagnosis, and guilt associated with smoking^14,15^, as well as healthcare system factors, such as the high financial cost of healthcare, lack of access to healthcare, and previous bad experiences with the healthcare system^15–18^.

Primary healthcare professionals (HCPs) play a key role in facilitating early diagnosis through recognising people with signs and symptoms suggestive of LC and referring them appropriately^19^. HCP-related barriers to early diagnosis of LC may include lack of awareness of signs and symptoms of LC, inadequate access to diagnostics and rapid referral pathways, and fear of overburdening the healthcare system^15,18^. In this systematic review, we identify and describe the effect of interventions aimed at helping HCPs recognise and refer individuals with signs and symptoms indicative of LC to the appropriate healthcare pathway in a timely manner.

## Methods

This systematic review was guided by the Cochrane Handbook for Systematic Reviews of Interventions^20^ and reported using the Preferred Reporting Items for Systematic Reviews and Meta-Analyses (PRISMA) checklist^21^ (Supplementary Table 1).

### Eligibility criteria

Using a modified version of the population, intervention, comparison, and outcomes (PICO) framework^22^, to include “S” for study design and “T” for timeframe (PICOST), the systematic review inclusion criteria were as follows: population: any HCPs. Studies were included only when patient outcomes were reported as a result of an intervention targeted towards HCPs; Intervention: any intervention, campaign, programme, trial, education, algorithm, decision tree/support, or guide aimed at improving early diagnosis of symptomatic LC; comparison: any pre-post comparison; outcomes: any outcomes (e.g., LC diagnosis among symptomatic patients, stage of LC at diagnosis, LC treatments received, and LC survival); study design: any experimental design; and timeframe: studies published between January 2011 and September 2021 in order to identify the latest evidence.

Studies were excluded if interventions were exclusively targeted at patients, did not incorporate a comparator, and/or used non-experimental designs. Studies focusing on detection of LC in asymptomatic individuals (i.e., through screening or surveillance) were also excluded. Moreover, we excluded conference proceedings, dissertations, and theses.

### Search strategy

MEDLINE, CINAHL, ERIC, and Academic Search Complete were searched on September 13, 2021. Truncation “*” was used and keywords were combined using Boolean operators “OR” and “AND” and the proximity indicator “N.” The following keywords were searched based on title or abstract: (Interven* OR program* OR campaign* OR trial* OR experiment* OR educat* OR algorithm* OR “decision* tree*” OR “decision* support*” OR guid*) AND (Refer* OR consult* OR recogni* OR counsel* OR advice OR advis* OR detect* OR find* OR triag* OR direct* OR manag* OR signpost* OR know* OR aware* OR understand*) AND ((Lung* OR pulmo*) N3 (cancer* OR neoplas* OR malignan* OR tumo* OR symptom* OR sign*)) AND (“Health* profession*” OR “health care profession*” OR HCP* OR “health* work*” OR “health care work*” OR HCW* OR clinician* OR nurs* OR “public health nurs*” OR PHN* OR “community nurs*” OR “clinic nurs*” OR “practice nurs*” OR pharmac* OR chemist* OR doctor* OR physician* OR “general practitioner*” OR GP* OR consultant*).

### Study extraction and synthesis

Records were screened in Covidence, an online software used to streamline the production of systematic reviews^23^. First, titles and abstracts were screened, and irrelevant records were excluded. Full texts of potentially eligible records were then sourced and screened. Each record was title, abstract, and full text screened twice by two independent reviewers. Screening conflicts were resolved by a third reviewer.

The following data were extracted for each study using a standardised table^14,24^ (Supplementary Table 2): author(s); year; country; aim; design; theoretical underpinning; sample; setting; relevant outcomes; intervention; procedures; instruments; follow-up time(s); and relevant findings. One reviewer conducted data extraction. Each extracted study was then cross-checked for accuracy by the review team. Meta-analyses were not plausible due to significant heterogeneity in study design, interventions, and outcome measures. Instead, a narrative synthesis was conducted, which involved grouping and synthesising the results according to the outcomes measured within the reviewed studies^25^.

### Quality appraisal and level of evidence assessment

The Mixed Methods Appraisal Tool was used to appraise the methodological quality of the included randomised controlled trials (RCTs) and non-RCTs^26^. Quality appraisal was conducted in terms of the appropriateness of recruitment, data collection, and data analysis to the research question. Each item was voted on a “yes,” “no,” and “cannot tell” basis. The Scottish Intercollegiate Guidelines Network^27^ grading system was used to assess the level of evidence for each of the included studies. The eight levels of evidence range between 1++, 1+, 1−, 2++, 2+, 2−, 3, and 4. For instance, a score of 1++ corresponds to high quality meta-analyses, systematic reviews of RCTs, or RCTs with a very low risk of bias, whereas a score of 4 is assigned to expert opinions^27^. Quality appraisal and level of evidence assessment were conducted by one reviewer and cross-checked for correctness by the review team.

## Results

### Study selection

**Fig. 1:**
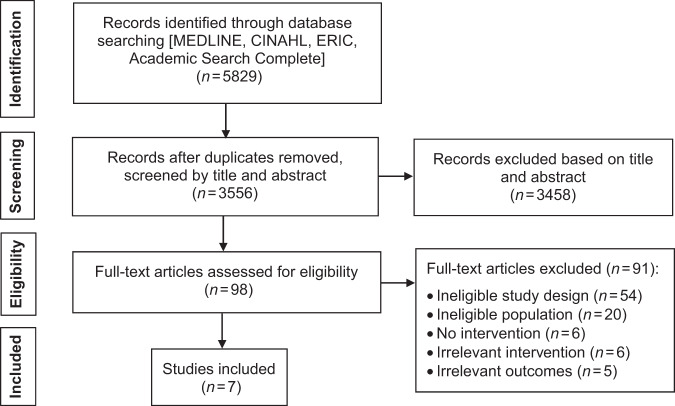
PRISMA flow diagram. Study identification, screening, and selection process.

Database searching resulted in 5829 records. Following deletion of duplicates, 3556 records were screened by title and abstract and 3458 irrelevant records were excluded. The full texts of the remaining 98 records were obtained and screened. Of those, seven were included in this systematic review (Fig. 1).

### Study characteristics

Most of the studies were conducted in Denmark (*n* = 2) and England (*n* = 2) and were non-RCTs (*n* = 5). Sample size ranged widely between 72^28^ and 56,020^29^ participants and follow-up times varied from 3^30^ to 37 months^31^. Five different interventions were used across the seven studies, including: (i) Combined public and HCP LC awareness campaigns;^30,32^ (ii) letters and continuing medical education (CME) meetings to educate general practitioners (GPs) about referral criteria for fast-track evaluation of patients with “reasonable suspicion” of LC (maximum 72 h waiting time for evaluation, which includes low dose computed tomography [LDCT]);^33,34^ (iii) a cancer fast-track programme (i.e., target of 30 days between well-founded suspicion of cancer by a GP and the start of treatment). Referrals to this programme can also originate from emergency departments or other clinical departments involved in routine monitoring or screening;^29^ (iv) the thoracic-trained advanced practice provider-led LC strategist programme to minimise diagnostic redundancy, streamline management decisions for indeterminate nodules, and expedite curative therapy. Once patients were referred from primary care to secondary care, an individual evaluation strategy was developed and followed for them;^31^ and (v) multi-disciplinary meetings, screensavers, and posters to reduce delay between initial suspicion of LC and measurement of serum calcium levels^28^. Of note, Hypercalcaemia is a serious complication of LC and is associated with poorer prognosis^28^. The full characteristics of the included studies are presented in Table 1.

**Table 1:** Study characteristics (*n* = 7).

Country	Denmark (*n* = 2)
	England (*n* = 2)
	Australia (*n* = 1)
	Spain (*n* = 1)
	United States of America (*n* = 1)
Research design	Non-randomised pre-post (*n* = 2)
	Randomised controlled trial (*n* = 2)
	Cohort (*n* = 1)
	Mixed method (*n* = 1)
	Retrospective (*n* = 1)
Sample size (min-max)	72–56,020
Settings	General practices and department of radiology in a university hospital (*n* = 2)
	Catalonian Health Service (*n* = 1)
	Community areas (*n* = 1)
	High-risk communities and GP surgeries (*n* = 1)
	Hospital (*n* = 1)
	Thoracic surgery clinic (*n* = 1)
Relevant outcomes^a^	Diagnostic intervals (*n* = 4)
	Referral and diagnosis patterns (*n* = 3)
	Stage distribution at diagnosis (*n* = 3)
	Time interval from diagnosis to treatment (*n* = 2)
Intervention	Combined public and healthcare professional awareness campaigns (*n* = 2)
	Letters and continuing medical education meetings (*n* = 2)
	Cancer fast track programme (*n* = 1)
	Multi-disciplinary meetings, screensavers, and posters (*n* = 1)
	Specialist-led LC strategist programme (*n* = 1)
Follow-up time (min-max)^b^	3–37 months

^a^Studies often reported on more than one outcome. n corresponds to the number of times an outcome was measured.

^b^One study did not report on length of follow-up.

### Quality appraisal and level of evidence assessment

All the included non-RCTs (*n* = 5) used appropriate data collection methods, outcome measures, and intervention administration. Outcome data were complete in all non-RCTs. Four non-RCTs had clear research questions. The study by Philips et al.^31^ did not have a clear aim statement, despite clearly stated hypotheses. Only one non-RCT reported that participants were representative of the target population^33^ and only one non-RCT reported that confounders were accounted for in the study design^28^. Both RCTs (*n* = 2) had clear research aims, performed randomisation appropriately, collected data in line with the research aims, had groups that were comparable at baseline, and reported on participant adherence to the assigned intervention^30,34^. However, the outcome assessor was not blinded in Gudlbrant et al.’s^34^ RCT.

Four studies scored 2+ on the Scottish Intercollegiate Guidelines Network^27^ level of evidence criteria, indicating well-conducted non-RCTs with a low risk of confounding or bias and a moderate probability that the relationship is causal^29,31–33^. Only one study scored 2++, indicating a well-conducted non-RCT with a low risk of confounding or bias and a moderate probability that the relationship is causal^28^. Both RCTs scored 1+ indicating well-conducted RCTs with a low risk of bias^30,34^. See Table 2 for quality and level of evidence assessment.

**Table 2:** Quality appraisal and level of evidence assessment.

Study designs	Author(s) & year	Quality appraisal items^a^												Level of evidence
		1	2	3	4	5	6	7	8	9	10	11	12	
Non-randomised studies^b^	Apthorp et al.^28^.	Y	Y	CT	Y	Y	Y	Y	–	–	–	–	–	2++
	Athey et al.^32^.	Y	Y	CT	Y	Y	N	Y	–	–	–	–	–	2+
	Guldbrant et al.^33^.	Y	Y	Y	Y	Y	CT	Y	–	–	–	–	–	2+
	Philips et al.^31^.	CT	Y	CT	Y	Y	CT	Y	–	–	–	–	–	2+
	Prades et al.^29^.	Y	Y	CT	Y	Y	CT	Y	–	–	–	–	–	2+
Randomised controlled trials^c^	Emery et al.^30^.	Y	Y	–	–	–	–	–	Y	Y	N	Y	Y	1+
	Gudlbrant et al.^34^.	Y	Y	–	–	–	–	–	Y	Y	Y	N	Y	1+

^a^All studies:

1 = clear research questions/aims 2 = data collected address research question/aims

^b^Non-randomised studies:

3 = participants representative of target population 4 = measurements appropriate regarding both the outcome and the intervention 5 = complete outcome data 6 = confounders accounted for in the design and analysis 7 = the intervention administered as intended

^c^Randomised controlled trials:

8 = randomisation appropriately performed 9=Groups comparable at baseline 10 = there are complete outcome data 11 = outcome assessors blinded to the intervention 12 = participants adhered to the assigned intervention

*CT* can’t tell, *N* no, *Y* yes.

### Synthesis of findings

Outcomes reported in the reviewed studies were categorised into four categories as follows: diagnostic intervals; referral and diagnosis patterns; stage distribution at diagnosis; and time interval from diagnosis to treatment.

**Table 3:** Data extraction and summary of findings (*n* = 7).

Reference	Design	Sample	Setting	Intervention	Findings
Apthorp et al.^28^	Pre-post quality improvement project	*n* = 72 patients	Hospital in England	MDT meetings, screensavers, and posters	Median delay 13 days pre-test vs 7 days post-test between referral to LC pathway and obtaining serum calcium levels (*p* = 0.001) MDT meetings: median delay 9 days between suspicion and investigation Posters: decreased delay of 6 days between suspicion and investigation Screensavers: decreased delay of 7 days between suspicion and investigation
Athey et al.^32^	Pre-post telephone survey	*n* = 1601 members of the public	High-risk communities and GP surgeries in England	Combined public and HCP LC awareness campaigns using a push-pull approach	Compared to 6 weeks pre-test and during campaign, x-ray referrals increased by 289 (22%). 169 more x-rays obtained (19% increase) in CG and 120 more x-rays in IG (27% increase) 12 months post-test: continued increase in chest x-rays requested in IG (extra 567 chest x-rays [20% increase]) vs 32 fewer x-rays (2% fall) in CG Significant increase in the number of chest x-rays over time between IG and CG (incidence rate ratio = 1.22, 95%CI 1.12–1.33, *p* = 0.001) LC diagnoses increased by 27% in IG and fell by 10% in CG 12 months post-test (not statically significant; incidence rate ratio = 1.42; 95%CI 0.83–2.44; *p* = 0.199) No significant stage shift 3 months, 6 months, or 1 year post-test
Emery et al.^30^	2 × 2 Factorial cluster RCT	*n* = 1358 participant	Community areas in Australia	Combined public and GP LC awareness campaign	Community intervention vs control: Median total diagnostic interval for LC 114.5 vs 114 days, mean difference=0.06, 95%CI 0.39–0.5, *p* = 0.79; GP intervention vs control: 115 vs 125 days; mean difference=0.02, 95%CI 0.56–0.60, *p* = 0.45
Guldbrandt et al.^33^	Cohort study nested in an RCT	*n* = 133 GPs	60 general practices and department of radiology in a university hospital in Denmark	Letters and CME meetings for GPs on referral criteria for LDCT	91 (68.4%) GPs used direct CTs Referral rate to direct CT significantly (61%) higher (95%CI 54-66%) among GPs working in a clinic with one or more CME-participating GPs 335 patients referred to LC fast-track. Of those, 33 (10%) had confirmed LC diagnosis. Of those, 8 (23.5%) had early-stage LC and 26 (76.5%) had advanced LC Referral rate to LC fast-track was 0.13 (95%CI 0.09 to 0.19) for CME-participating GPs vs 0.14 (95%CI 0.09–0.20) for non-participating GPs (*p* = 0.503) PPV for LC diagnosis due to referral to fast-track LC pathway 13.3% (95%CI 8.7 to 19.1%) for CME-participating GPs vs 6.1% (95%CI 3–11%) for non-participating GPs (*p* = 0.027; i.e., 2.2 higher PPV)
Guldbrandt et al.^34^	Cluster RCT	*n* = 266 GPs	119 general practices and department of radiology in a university hospital in Denmark	Letters and CME meetings for GPs on referral criteria for LDCT	No statistically significant difference in primary care interval between patients in IG (media *n* = 14 days, IQI = 4–53) and patients in CG (media *n* = 18 days, IQI = 5–69, prevalence ratio=0.99, 95%CI 0.65–1.54, *p* = 0.455) No statistically significant difference in diagnostic interval between patients in IG (media *n* = 44 days, IQI = 1783) and patients in CG (media*n* = 36 days, IQI = 17–112, PR = 0.8, 95%CI 0.5–1.27, *p* = 0.299) Primary care and diagnostic intervals shorter for CME-participating GPs (primary care interval median = 9 days [with CME] vs 37 days [without CME], *p* = 0.048; diagnostic interval median = 23 days [with CME] vs 66 days [without CME], *p* = 0.008) Non-statistically significant higher risk of having a long diagnostic interval for patients in the CG (risk difference = 13.5%, 95%CI -11–37.9%, *p* = 0.280). No statistically significant difference in risk for having a long primary care interval was observed using this approach (risk difference = 1.1%, 95%CI 23.9–26.1%, *p* = 0.929) Non-statistically significant difference in stage of LC at diagnosis for all patients (*p* = 0.586 for advanced [stage IV] LC and *p* = 0.595 for localised [stage IA-IIIA] LC) Non-statistically significant difference in stage of LC at diagnosis for patients whose GP was involved in the diagnosis (*p* = 0.47 for advanced [stage IV] LC and *p* = 0.658 for localised [stage IA-IIIA] LC)
Philips et al.^31^	Retrospective review	*n* = 200 patients	Thoracic surgery clinic in the USA	Thoracic-trained advanced practice provider-led LC strategist programme	Time from suspicion to workup significantly shorter in the programme vs routine referral (3 vs 28 days, *p* < 0.001). Following referral, median time to workup significantly shorter in the programme vs routine referral (1 vs 7 days, *p* < 0.001) Time from suspicion to management plan 14.5 days in the programme vs 46.5 days in routine referral (*p* < 0.001) Programme referral moved patients into surveillance a month earlier vs routine referral (12.5 vs 39 days, *p* < 0.001). Following designation to surveillance, 33 programme (42.3%) and 9 routine referrals (21.9%) were discharged early. Management via programme reduced the median number of hospital trips (4 vs 6, *p* < 0.001), clinicians seen (1.5 vs 2, *p* = 0.08), diagnostic studies (4 vs 5, *p* = 0.01), time from suspicion to diagnosis (30.5 vs 48, *p* = 0.02), and treatment (40.5 vs 68.5, *p* = 0.02) Time to surgery significantly shorter in patients managed by the programme vs routine referral (38 vs 69 days, *p* = 0.05). Among patients with early-stage non-small cell LC treated with radiotherapy, the programme led to a substantial reduction in the time from suspicious finding to initiation of treatment in comparison to routine referral (62.5 vs 122.5 days, *p* = 0.08). No significant difference in stage at diagnosis for the 7 programme and 33 routine referral patients who underwent surgery for non-small cell LC. 6 of 7 LCSP patients (85.7%) had early-stage LC, with a median time from suspicion to treatment of 37 days. In these 6 patients, disease free and overall survival were 100%, with a median duration of follow up of 33 months. In routine referral, 25 of 33 patients (75.7%) had early-stage disease, with a median time from suspicion to treatment of 68 days. In these 25 patients, there have been 6 recurrences (disease-free survival = 76%) and no deaths (overall survival = 100%) with a median duration of follow up of 35 months.
Prades et al.^29^	Mixed methods	*n* = 56,020 individuals	Catalonian Health Service in Spain	Cancer fast-track programme	Decrease in the proportion of patients diagnosed through the programme from 2006 (60.2% [95%CI 59.8–63.4%]) to 2009 (53.2% [95%CI 51.5–54.9%]) Decrease in the proportion of LC patients referred by a GP from 2006 (60.6% [95%CI 59–62.3%]) to 2009 (41.4% [95%CI 39.7–42.9%]) LC detection rate decreased from 49.9% (95%CI 48.2–51.6) in 2006 to 39.7% (95%CI 38.1–41.2%) in 2009 Mean time from detection of suspected LC in primary care to start of treatment increased from 30.8 days (2006) to 36.7 days (2009) Increase in proportion of LC patients waiting over 45 days from the time of detection of suspected cancer to start of treatment (13.6% in 2006 vs 22.6% in 2009) Increase in proportion of LC cases waiting between 30 and 45 days (23.7% in 2006 to 26.1% in 2009) Increase in compliance with referral guidelines from 70.8% in 2006 (95%CI 69.1–72.1%) to 82.3% in 2009 (95%CI 81.1–83.5%)

*CG* control group, *CI* confidence interval, *CME* continuing medical education, *CT* computed tomography, *GP* general practitioner, *HCP* healthcare professional, *IG* intervention group, *IQI* inter quartile interval, *LC* lung cancer, *LDCT* low dose computed tomography, *MDT* multidisciplinary team, *PPV* positive predictive value, *RCT* randomised controlled trial, *USA* United States of America.

The data extraction table is presented in Table 3 and findings from this review are summarised in Table 4.

**Table 4:** Visual representation of the key review findings.

Reference	Intervention	Outcomes			
		Diagnostic interval	Referral and diagnosis patterns	Stage distribution at diagnosis	Time interval from diagnosis to treatment
Apthorp et al.^28^	MDT meetings, screensavers, and posters	↑	–	–	–
Athey et al.^32^	Push-pull public and HCP LC awareness campaigns	–	↑	↔	–
Emery et al.^30^	Public and GP LC awareness campaign	↔		–	–
Guldbrandt et al.^33^	Letters and CME meetings for GPs on referral criteria for LDCT	–	↑	–	–
Guldbrandt et al.^34^	Letters and CME meetings for GPs on referral criteria for LDCT	↔^a^ ↑	–	↔	–
Philips et al.^31^	Advanced practice provider-led LC strategist programme	↑	–	↔	↑
Prades et al.^29^	Cancer fast-track programme	–	↓	–	↓^b^

↑ Significant improvement.

↔ No significant difference.

↓ Significant deterioration.

^a^No statistically significant difference between the intervention group and the control group. However, the primary care and diagnostic intervals in the intervention group were significantly shorter if GPs participated in CME.

^b^Unclear whether this finding was statistically significant.

*CME* continuing medical education, *GP* general practitioner, *HCP* healthcare professional, *LC* lung cancer, *LDCT* low dose computed tomography, *MDT* multidisciplinary team.

#### Diagnostic interval

Four studies aimed to reduce the diagnostic interval (i.e., the time from the first presentation with symptoms of LC until diagnosis^35^) using the LC strategist programme^31^, a community- and GP-targeted cancer awareness campaign^30^, information on LDCT and CME sessions^34^, and a multimodal quality improvement project^28^.

A retrospective review of the LC strategist programme found that time from suspicious findings on CT chest, chest X-ray, and to a lesser extent abdominal CT, to initiation of diagnostic workup of lung nodules for treatment or surveillance was significantly shorter with the programme in comparison to routine referral (3 vs 28 days respectively, *p* < 0.001)^31^. Following referral, the median time to workup was also significantly shorter with the programme in comparison to routine referral (1 vs 7 days respectively, *p* < 0.001)^31^.

In contrast, a concurrent community- and GP-targeted breast, prostate, colorectal, and LC awareness campaign found no statistically significant difference in the total diagnostic interval at community (i.e., public intervention) level (median total diagnostic interval = 114.5 days pre-test vs 114 days post-test, mean difference = 0.06, 95% confidence interval [CI] 0.39–0.5, *p* = 0.79) or at GP level (median total diagnostic interval = 115 days pre-test vs 125 days post-test, mean difference = 0.02, 95%CI 0.56–0.60, *p* = 0.45)^30^. Likewise, a study measuring the effect of an intervention to inform GPs about direct access to LDCT found no statistically significant difference in primary care interval (i.e., the time from the patient’s first symptomatic presentation in primary care until referral to secondary care^35^) between patients of GPs who received information about indications for LDCT (intervention group) (media *n* = 14 days, inter quartile intervals [IQI] = 4–53) and patients of GPs who did not receive this information (control group) (media *n* = 18 days, IQI = 5–69, Prevalence Ratio [PR] = 0.99, 95%CI 0.65–1.54, *p* = 0.455)^34^. Moreover, no statistically significant difference was found in the diagnostic interval between patients in the intervention group (media*n* = 44 days, IQI = 17–83) and the control group (media *n* = 36 days, IQI = 17-112, PR = 0.8, 95%CI 0.5–1.27, *p* = 0.299). However, the primary care and diagnostic intervals in the intervention group were significantly shorter if the GP also participated in a 1-h small-group-based CME session (primary care interval median = 9 days [with CME] vs 37 days [without CME], *p* = 0.048; diagnostic interval median=23 days [with CME] vs 66 days [without CME], *p* = 0.008)^34^.

In their quality improvement project, Apthrop et al.^28^ used multidisciplinary meetings, screensavers, and posters encouraging secondary care physicians to order serum calcium levels in patients with a suspected diagnosis of LC. This project aimed to help reduce delay between initial suspicion of LC and ordering serum calcium levels during initial LC diagnostic workup in England. This project led to a statistically significant reduction in overall median time to ordering serum calcium levels in patients with a suspected diagnosis of LC, from 13 days pre-test (i.e., before the quality improvement project) to 7 days post-test (*p* = 0.001)^28^.

#### Referral and diagnosis patterns

Three studies reported on patterns of LC referral and diagnosis following implementation of a public awareness and GP training campaign^32^, a cancer fast-track programme^29^, and GP information and CME sessions on indications for LDCT^33^. Athey et al.^32^ delivered a public and GP LC awareness campaign in six English communities with high LC incidence served by 11 GP surgeries (intervention group). This campaign ran for six weeks and used a “push-pull” approach to “push” the public to seek help for symptoms of concern and encourage GPs to “pull” symptomatic individuals into appropriate services. Five other communities served by nine GP surgeries with similar demographics served as the control group. There was a 27% increase in the number of chest X-rays ordered in the intervention group compared to a 19% increase in the control group during the campaign and six months post-test. In comparison to pre-campaign, there was a sustained increase in chest X-rays requested in the intervention group (20% relative increase) in comparison to a 2% relative reduction in the control group (Incidence Rate Ratio [IRR] = 1.22, 95%CI 1.12–1.33, *p* = 0.001) at 12 months post-campaign. Moreover, LC diagnoses increased by 27% (relative increase) in the intervention group and fell by 10% (relative reduction) in the control group. However, this was not statistically significant (IRR = 1.42, 95%CI 0.83–2.44, *p* = 0.199)^32^.

In a study of a cancer fast-track programme in Catalonia, Prades et al.^29^ noted increased use of the programme over time, with 3336 patients with suspected LC referred via the programme in 2006, compared to 3841 patients in 2009. The proportion of all new LCs that were diagnosed through this programme fell from 60.2% (95%CI 59.8–63.4%) in 2006 to 53.2% (95%CI 51.5–54.9%) in 2009. GPs were the source of 60.6% of referrals to the fast-track programme in 2006 (95%CI 59–62.3%), falling to 41.4% (95%CI 39.7–42.9%) in 2009, demonstrating increased referrals from other sources such as hospital-based clinicians and services. The LC detection rate via the programme fell from 49.9% (95%CI 48.2–51.6%) in 2006 to 39.7% (95%CI 38.1–41.2%) in 2009. Prades et al.^29^ reported a statistically significant increase in GP compliance with cancer fast-track referral guidelines from 70.8% in 2006 (95%CI 69.1–72.1%) to 82.3% in 2009 (95%CI 81.1–83.5%).

In a cohort study nested in an RCT, Guldbrandt^33^ examined the use of a fast-track referral option for GPs for patients with suspected LC and the effect of GP education and awareness training on direct referral to LDCT. This education comprised a one-hour CME session and information about LDCT, including indications and Positive Predictive Values (PPV) for LC (i.e., the ratio of patients truly diagnosed as positive to all those who had positive test results). Results showed that, out of 648 patients directly referred to LDCT, absolute numbers of referrals were significantly higher (61%, 95%CI 54–66%) among GPs working in a clinic with one or more CME-participating GPs. However, the referral rate to LDCT via fast-track was 0.13 per 1000 adults per month (95%CI 0.09–0.19) for CME-participating GPs compared to 0.14 (95%CI 0.09–0.20) for non-participating GPs. The PPV for LC diagnosis due to referral to a fast-track LC pathway was 13.3% (95%CI 8.7–19.1%) for CME-participating GPs and 6.1% (95%CI 3–11%) for non-participating GPs (2.2 higher PPV). This was found to be statistically significant (*p* = 0.027)^33^.

#### Stage distribution at diagnosis

Three studies reported on LC stage at diagnosis following an intervention. Athey et al.^32^ examined LC stage at diagnosis following a “push-pull” LC awareness campaign, Guldbrandt et al.^34^ examined LC stage at diagnosis following an information programme and CME sessions on LDCT for GPs, and Philips et al.^31^ examined LC stage at diagnosis following the LC strategist programme. Athey et al.^32^ found no significant stage shift three months, six months, or one year following the LC “push-pull” awareness campaign. Similarly, Guldbrandt et al.^34^ reported a non-statistically significant difference in stage of LC at diagnosis between the intervention group (i.e., information and CME sessions on LDCT) and control group (*p* = 0.586 for advanced LC and *p* = 0.595 for localised LC). Philips et al.^31^ also found non-statistically significant difference in stage at diagnosis for the seven patients in the LC strategist programme and 33 routine referral patients who underwent surgery for LC. This was the only study to report on disease free survival and overall survival. It was found that six of the seven patients (85.7%) in the LC strategist programme cohort were found to have early-stage disease with a median time of 37 days from suspicious imaging to treatment^31^. In these six patients, with a median duration of follow up of 33 months, disease free survival and overall survival were 100% (i.e., no LC recurrence and no LC death). As for the routine referral group, 25 of 33 patients (75.7%) were found to have early-stage LC with a median time of 68 days from suspicious imaging to treatment. In these 25 patients, there were six recurrences (76% disease free survival) and no deaths (100% overall survival) over a median time of 35 months. The differences in survival rates between the LC strategist programme group and the routine referral group were not statistically significant^31^.

#### Time interval from diagnosis to treatment

The time from LC diagnosis to treatment was measured in two studies following two specialist programmes, namely the cancer fast-track programme^29^ and the LC strategist programme^31^. The latter study found that the time from suspicious imaging to definitive management plan was 14.5 days in the LC strategist programme and 46.5 days in routine referral (*p* < 0.001)^31^. It was also found that referral to the programme moved patients into low-risk nodule surveillance approximately one month earlier relative to routine referral (12.5 vs 39 days respectively, *p* < 0.001). Compared to routine referral, management through the programme also significantly reduced the median number of hospital trips (4 vs 6 respectively, *p* < 0.001), median number of clinicians seen (1.5 vs 2 respectively, *p* = 0.08), median number of diagnostic studies obtained (4 vs 5 respectively, *p* = 0.01), median time from suspicious radiological findings to diagnosis (30.5 vs 48 days respectively, *p* = 0.02), and median time from suspicious radiological findings to treatment (40.5 vs 68.5 days respectively, *p* = 0.02)^31^. Moreover, time from suspicious radiological findings to surgical resection was significantly shorter in patients managed through the programme in comparison to routine referral (38 vs 69 days respectively, *p* = 0.05). Among patients with early-stage non-small cell LC treated with radiation therapy, the LC strategist programme led to a substantial reduction in the time from suspicious radiological findings to initiation of treatment in comparison to routine referral (62.5 vs 122.5 days respectively, *p* = 0.08)^31^. Conversely, in the cancer fast-track programme, Prades et al.^29^ noted a variable trend in mean time from detection of suspected LC in primary care to start of initial treatment. The 30-day target was not achieved, with mean times of 30.8 days, 38.9 days, 32.25 days, and 36.7 days in 2006, 2007, 2008, and 2009 respectively. There was also an increase in the proportion of patients waiting between 30 and 45 days (23.7% in 2006 vs 26.1% in 2009) and over 45 days (13.6% in 2006 vs 22.6% in 2009) from the time of LC detection to initiation of treatment.

## Discussion

Achieving early diagnosis is an essential step in improving LC outcomes^28–31,34^. While more than 85% of patients subsequently diagnosed with cancer initiate their diagnostic pathway in primary care^35^, timely recognition and referral of people with suspected LC is complicated by various primary HCP and system-related factors. For example, a scoping review of 33 studies identified low index of suspicion, delays in obtaining access to diagnostic tests, multiple specialist consultations and lack of rapid assessment services as barriers to early diagnosis of LC^36^. Additionally, a qualitative study of 16 GPs from five practices in the United Kingdom found that GPs often required high levels of suspicion to refer patients to secondary care and were concerned about overloading the healthcare system by over-referring patients^37^. More recently, Saab et al.^38^ interviewed 36 primary HCPs (GPs, community pharmacists, GP practice nurses, and public health nurses) about their experience of referring individuals with suspected LC in Ireland. It was found that “typical” LC lung signs and symptoms such as cough and haemoptysis triggered referrals, whereas “atypical” signs and symptoms like back pain and pallor, were perceived as difficult to interpret. Participants suggested educating primary HCPs about early LC referral using “communications from professional organisations, webinars, interdisciplinary meetings, education by lung specialists, and patient testimonials” (p.1)^38^. The use of simple, clear, and visually appealing LC referral checklists and algorithms in primary care was also recommended^38^.

Several studies included in the present review reported on efforts to raise awareness of LC signs and symptoms among HCPs, and prompt timely referral for further diagnostic or specialist evaluation. These included: a combined public and HCP LC awareness campaign which used GP education resource cards with symptom risk assessment charts to increase symptom awareness and early specialist referral among GPs;^30^ a push-pull campaign that involved educating GPs and community pharmacists about chest X-ray referral criteria for symptomatic patients;^32^ and CME sessions for GPs addressing the indications for LDCT for signs and symptoms that raised GPs’ suspicion of LC, but fell short of satisfying the fast-track referral criteria^33,34^. Indeed, the effect of CME meetings on raising GPs’ awareness of cancer signs and symptoms and prompting early referral is well documented in the wider literature. Toftegaard et al.^39^ studied the impact of CME meetings in Denmark to support GPs in recognising and referring patients with cancer warning signs and symptoms. An evaluation of this initiative found that CME meetings significantly improved knowledge of cancer among GPs and increased the number of urgent referrals^39^, which is associated with better cancer survival^40,41^.

Interventions that were successful in reducing the diagnostic interval included a multi-modal quality improvement project in primary care^28^ and the LC strategist programme in secondary care^31^. In contrast, statistically significant reductions in diagnostic intervals were not achieved following a community- and GP-targeted awareness campaign^30^ as well as information for GPs on LDCT for symptomatic patients^34^. GP participation in a 1-h CME session on LDCT, however, was associated with shorter primary care and diagnostic intervals^34^, higher absolute number of referrals to LC fast-track, and higher PPV for LC diagnosis^33^.

Postal questionnaires offer a pro-active, if somewhat resource intensive, option for primary HCPs to prompt help-seeking among high-risk symptomatic patients. For example, Wagland et al.^42^ studied the impact of sending a postal symptom questionnaire, incorporating nine symptoms of LC, to patients identified as high risk for LC in eight GP practices in England. Through this intervention, a small, clinically relevant group (6.7%, *n* = 61/908) of primary care patients was identified who, despite reporting potential symptoms of LC, had not consulted a GP in ≥12 months. Primary care consultations significantly increased in the 3-month period following receipt of the symptom elicitation questionnaire compared to the 3-month period pre-questionnaire (*p* = 0.002)^42^. Participants who decided not to consult their GP cited concerns over wasting their own and the GP time and reported a high symptom tolerance threshold and a greater tendency to self-manage their symptoms^42^. These barriers are well documented in the wider literature^15,16,18^.

The benefits of cancer fast-track pathways/programmes are well documented in the international literature^43–46^. Fast-track referral criteria are typically based on the presence of combinations of, or individual, ‘alarm’ cancer signs and symptoms and/or relevant radiological findings, usually with a PPV for cancer of 3% or above^47^. Two of the reviewed studies evaluated the impact of specialist-led and fast-track programmes on time from suspicious radiologic findings^31^ and LC detection^29^ to the planning and initiation of treatment. In comparison to routine referral, the specialist-led LC strategist programme significantly reduced the intervals between suspicious radiologic findings and definitive management plan, diagnosis, and treatment^31^. In contrast, in their evaluation of a cancer fast-track programme from its inception in 2006 until 2009, Prades et al.^29^ reported a significant increase in waiting times from LC detection to initiation of treatment. This may be explained by factors including the complexity of LC treatment, including thoracic surgery at tertiary hospitals^29^.

Interventions aimed at prompting early referral and diagnostic work-up do not always lead to significant improvements in stage of LC at diagnosis and overall survival. Our systematic review demonstrated that CME sessions on the indications for LDCT^34^, the specialist-led LC strategist programme^31^, and a combined public and HCP cancer awareness campaign^32^, were not associated with significant differences in stage of LC at diagnosis. In addition, Philips et al.^31^ found non-statistically significant differences in LC recurrence and mortality in patients referred through the LC strategist programme in comparison to those referred through routine referral. Larger scale studies with more statistical power and prospective RCTs with longer follow-up are recommended^31,32,34^.

This review offers valuable insights into interventions aimed at improving the early diagnosis of symptomatic LC. However, a few limitations are worthy of note. While there is some evidence for the effectiveness of CME meetings and fast-track programmes, recommendations for clinical practice should be made with caution, particularly due to the small number of studies included in this review and the fact that meta-analyses were not possible due to significant heterogeneity in study design, interventions, and outcome measures. Study selection bias could have occurred, as only studies relevant to the review aims were included, the search did not include records from the grey literature or clinical trial registries, and the review was limited to studies published within a 10-year timeframe.

In conclusion, findings from this review indicate that CME meetings for primary HCPs may facilitate early LC referral, diagnosis, and survival. We also found evidence that fast-track programmes, such as the LC strategist programme^31^, may improve time from initial presentation with symptoms in primary care to LC diagnosis, and time from diagnosis to treatment, in addition to reducing hospital visits and the number of clinicians seen between initial presentation and initiation of treatment. However other interventions, such as awareness campaigns, were not associated with significant improvements in outcomes^30,32^. Outcomes such as LC stage shift and mortality rates were seldom measured in the reviewed studies. When measured, statistical significance was not reached, hence the importance of conducting future studies that are appropriately powered, controlled, and have longer follow-up.

Review findings may inform cancer control policy, including the design and implementation of interventions aimed at overcoming barriers to early LC diagnosis. These interventions may include awareness and education campaigns targeting the public and HCPs, and implementation of specialist-led fast-track referral programmes to facilitate timely diagnosis.

## Supplementary information

Supplementary Tables 1 and 2

## Data Availability

Data sharing not applicable to this article as no datasets were generated or analysed during the current study.
