# Supplementary material for: A systematic review of interventions to recognise, refer and diagnose patients with lung cancer symptoms
Source: NPJ Prim Care Respir Med. 2022 Oct 18;32:42. doi: 10.1038/s41533-022-00312-9 (PMC9579201; doi:10.1038/s41533-022-00312-9)
Supplement: Supplementary file 1 — Supplementary Tables 1 and 2 [file 41533_2022_312_MOESM1_ESM.docx]

**Supplementary Table 1.** PRISMA 2020 Checklist

| **Section and Topic** | **Item #** | **Checklist item** | **Location where item is reported** |
| --- | --- | --- | --- |
| **TITLE** | | |  |
| Title | 1 | Identify the report as a systematic review. | Page 1 |
| **ABSTRACT** | | |  |
| Abstract | 2 | See the PRISMA 2020 for Abstracts checklist. | Page 2 |
| **INTRODUCTION** | | |  |
| Rationale | 3 | Describe the rationale for the review in the context of existing knowledge. | Pages 3 and 4 |
| Objectives | 4 | Provide an explicit statement of the objective(s) or question(s) the review addresses. | Page 4 |
| **METHODS** | | |  |
| Eligibility criteria | 5 | Specify the inclusion and exclusion criteria for the review and how studies were grouped for the syntheses. | Page 4 |
| Information sources | 6 | Specify all databases, registers, websites, organisations, reference lists and other sources searched or consulted to identify studies. Specify the date when each source was last searched or consulted. | Page 5 |
| Search strategy | 7 | Present the full search strategies for all databases, registers and websites, including any filters and limits used. | Page 5 |
| Selection process | 8 | Specify the methods used to decide whether a study met the inclusion criteria of the review, including how many reviewers screened each record and each report retrieved, whether they worked independently, and if applicable, details of automation tools used in the process. | Page 5 |
| Data collection process | 9 | Specify the methods used to collect data from reports, including how many reviewers collected data from each report, whether they worked independently, any processes for obtaining or confirming data from study investigators, and if applicable, details of automation tools used in the process. | Pages 5 and 6 |
| Data items | 10a | List and define all outcomes for which data were sought. Specify whether all results that were compatible with each outcome domain in each study were sought (e.g. for all measures, time points, analyses), and if not, the methods used to decide which results to collect. | Pages 5 and 6 |
|  | 10b | List and define all other variables for which data were sought (e.g. participant and intervention characteristics, funding sources). Describe any assumptions made about any missing or unclear information. | Pages 5 and 6 |
| Study risk of bias assessment | 11 | Specify the methods used to assess risk of bias in the included studies, including details of the tool(s) used, how many reviewers assessed each study and whether they worked independently, and if applicable, details of automation tools used in the process. | Page 6 |
| Effect measures | 12 | Specify for each outcome the effect measure(s) (e.g. risk ratio, mean difference) used in the synthesis or presentation of results. | N/A |
| Synthesis methods | 13a | Describe the processes used to decide which studies were eligible for each synthesis (e.g. tabulating the study intervention characteristics and comparing against the planned groups for each synthesis (item #5)). | Pages 5 and 6 |
|  | 13b | Describe any methods required to prepare the data for presentation or synthesis, such as handling of missing summary statistics, or data conversions. | N/A |
|  | 13c | Describe any methods used to tabulate or visually display results of individual studies and syntheses. | Pages 5 and 6 |
|  | 13d | Describe any methods used to synthesize results and provide a rationale for the choice(s). If meta-analysis was performed, describe the model(s), method(s) to identify the presence and extent of statistical heterogeneity, and software package(s) used. | Pages 5 and 6 |
|  | 13e | Describe any methods used to explore possible causes of heterogeneity among study results (e.g. subgroup analysis, meta-regression). | Pages 5 and 6 |
|  | 13f | Describe any sensitivity analyses conducted to assess robustness of the synthesized results. | N/A |
| Reporting bias assessment | 14 | Describe any methods used to assess risk of bias due to missing results in a synthesis (arising from reporting biases). | N/A |
| Certainty assessment | 15 | Describe any methods used to assess certainty (or confidence) in the body of evidence for an outcome. | N/A |
| **RESULTS** | | |  |
| Study selection | 16a | Describe the results of the search and selection process, from the number of records identified in the search to the number of studies included in the review, ideally using a flow diagram. | Page 6 and Figure 1 |
|  | 16b | Cite studies that might appear to meet the inclusion criteria, but which were excluded, and explain why they were excluded. | Figure 1 |
| Study characteristics | 17 | Cite each included study and present its characteristics. | Pages 6 and 7 and Table 1 |
| Risk of bias in studies | 18 | Present assessments of risk of bias for each included study. | Pages 7 and 8 and Table 2 |
| Results of individual studies | 19 | For all outcomes, present, for each study: (a) summary statistics for each group (where appropriate) and (b) an effect estimate and its precision (e.g. confidence/credible interval), ideally using structured tables or plots. | Table 3, Table 4 and Supplementary Table 2 |
| Results of syntheses | 20a | For each synthesis, briefly summarise the characteristics and risk of bias among contributing studies. | Pages 8 to 13, Table 3, Table 4 and Supplementary Table 2 |
|  | 20b | Present results of all statistical syntheses conducted. If meta-analysis was done, present for each the summary estimate and its precision (e.g. confidence/credible interval) and measures of statistical heterogeneity. If comparing groups, describe the direction of the effect. | Pages 8 to 13, Table 3, Table 4 and Supplementary Table 2 |
|  | 20c | Present results of all investigations of possible causes of heterogeneity among study results. | Pages 8 to 13, Table 3, Table 4 and Supplementary Table 2 |
|  | 20d | Present results of all sensitivity analyses conducted to assess the robustness of the synthesized results. | N/A |
| Reporting biases | 21 | Present assessments of risk of bias due to missing results (arising from reporting biases) for each synthesis assessed. | Pages 7 and 8 |
| Certainty of evidence | 22 | Present assessments of certainty (or confidence) in the body of evidence for each outcome assessed. | N/A |
| **DISCUSSION** | | |  |
| Discussion | 23a | Provide a general interpretation of the results in the context of other evidence. | Pages 13 to 16 |
|  | 23b | Discuss any limitations of the evidence included in the review. | Page 16 |
|  | 23c | Discuss any limitations of the review processes used. | Page 16 |
|  | 23d | Discuss implications of the results for practice, policy, and future research. | Pages 16 and 17 |
| **OTHER INFORMATION** | | |  |
| Registration and protocol | 24a | Provide registration information for the review, including register name and registration number, or state that the review was not registered. | N/A |
|  | 24b | Indicate where the review protocol can be accessed, or state that a protocol was not prepared. | N/A |
|  | 24c | Describe and explain any amendments to information provided at registration or in the protocol. | N/A |
| Support | 25 | Describe sources of financial or non-financial support for the review, and the role of the funders or sponsors in the review. | Page 18 |
| Competing interests | 26 | Declare any competing interests of review authors. | Page 18 |
| Availability of data, code and other materials | 27 | Report which of the following are publicly available and where they can be found: template data collection forms; data extracted from included studies; data used for all analyses; analytic code; any other materials used in the review. | Page 18 and Supplementary Table 2 |

*From:*  Page MJ, McKenzie JE, Bossuyt PM, Boutron I, Hoffmann TC, Mulrow CD, et al. The PRISMA 2020 statement: an updated guideline for reporting systematic reviews. BMJ 2021;372:n71. doi: 10.1136/bmj.n71

For more information, visit: <http://www.prisma-statement.org/>

**Supplemental Table 2.** Data extraction table (n=7)

| **Author(s), Year & Country** | **Aim** | **Design & Theory** | **Sample & setting** | **Relevant Outcomes** | **Intervention** | **Procedures & Instruments** | **Follow-up time(s)** | **Relevant Findings** |
| --- | --- | --- | --- | --- | --- | --- | --- | --- |
| Apthorp et al. (2021)  England | To reduce delay between initial suspicion of lung cancer (LC) and serum calcium level measured as part of initial workup to improve patient outcomes | Pre-post quality improvement project | n=72 patients recruited from 5 weekly lung multidisciplinary team (MDT) meetings  Hospital | Dates of referral to LC team  Calcium and alanine aminotransferase (ALT) levels  Time between standard blood test, ALT, and adjusted serum calcium blood test | Interventions conducted over 9 months to evaluate which would have the largest impact.  Findings presented at MDT meetings to encourage the department's  participation in ordering calcium as part of initial workup for patients with suspected LC  Computers used to display data of the study via weekly screensaver bulletin  Posters displayed in the hospital to highlight results and encourage physicians to order serum calcium levels | Data collected via e-records system, obtaining date of referral to the LC team, and the adjusted calcium and ALT levels  Patients referred to the LC team with calcium and ALT checked within 4 weeks prior to the initial suspicion of LC were accepted to the study and recorded to have a 0-day interval between referral and blood investigations | 12 months | Median delay of 13 days pre-test vs 7 days post-test between initial referral to LC pathway and obtaining serum calcium levels (p=0.001)  Median delay of 9 days between suspicion of LC and investigation following MDT meetings  Posters led to a decreased delay of 6 days between suspicion of LC and investigation  Data presented on the trust screensavers led to a decreased delay of 7 days between suspicion of LC and investigation. |
| Athey et al. 2012)  England | To evaluate the effectiveness of a mixed method community based social marketing intervention on LC diagnosis | Pre-post telephone survey | n=1,601 members of the public (n=801 pre-test and 800 post-test) from 6 priority communities served  by 11 General Practitioner (GP) surgeries (Intervention Group [IG]) and 5 communities served by nine GP surgeries (Control Group [CG])  Community and GP surgeries | Chest x-ray rates  LC diagnosis  Stage at diagnosis | Push-pull approach.  *Push:* public awareness campaign designed by creative, media, and public relations agencies; face-to-face events; and conversations that focused on raising awareness of the importance of seeking medical advice and requesting a chest  x-ray for a cough lasting more than 3 weeks  *Pull:* Training HCPs (HCPs) for the initiative i.e., sharing insights, training, and capacity management in GP surgeries. HCPs reminded of National Institute for Health and Care Excellence chest x-ray referral criteria. Community pharmacists encouraged to promote campaign materials to patients buying over-the counter cough medication. GP practices visited and training delivered prior to the public campaign | Public awareness campaign evaluated by a telephone survey  Retrospective chest-x-ray data from the Radiology Information System. The numbers of x-rays requested by the practices over the 6 weeks before and after the interventions were recorded. These were compared with the GP x-ray request rates. Data compared between 12 months pre-test and 12 months post-test | 12 months | Compared to 6 weeks pre-test and during campaign, chest x-ray referrals increased by 289 (22%). 169 more x-rays obtained (19% increase) in CG and 120 more x-rays in IG (27% increase)  12 months post-test: continued increase in chest x-rays requested in IG (extra 567 chest x-rays [20% increase]) vs 32 fewer x-rays (2% fall) in CG  Statistically significant increase in the number of chest x-rays over time between IG and CG (Incidence Rate Ratio [IRR]=1.22, 95%Confidence Interval [CI] 1.12-1.33, p=0.001)  Compared with 12 months pre-test, LC diagnoses increased by 27% in IG and fell by 10% in CG. This was not statically significant (IRR=1.42; 95%CI 0.83-2.44; p=0.199)  No significant stage shift found at 3 months, 6 months, or 1 year post-test |
| Emery et al. (2017)  Australia | To measure the effect of community-based symptom awareness and general practice-based educational interventions on the time to diagnosis in rural patients presenting with breast,  prostate, colorectal or LC | 2x2 Factorial cluster randomised controlled trial (RCT)  The Model of Pathways to Treatment | n=1,358 participants with breast, prostate, colorectal, and LC (of those, n=117 participants had LC) from Trial Area A and Trial Area B  Community | Total Diagnostic Interval (TDI) i.e., time from first symptom to cancer diagnosis | *Community Intervention:* Modified “Find Cancer Early” United Kingdom-based campaign tailored for rural Australians.  *GP intervention:*  GP education resource card with symptom risk assessment charts and local cancer referral pathways created and implemented through multiple academic visits, to promote earlier recognition and investigation of suspicious symptoms by GPs and clarifying cancer | Trial Area A received the community symptom awareness campaign and Trial Area B acted as the community campaign control region. Within both Trial Areas, general practices were randomised to the GP intervention or control.  SYMPTOM questionnaire and GP record audit tool were used to calculate TDI:  *SYMPTOM questionnaire:* Participants answered items specific to LC to capture details of symptoms, their date of onset and time taken to seek help  *GP record audit tool:* Captured information on the date, type and duration of symptoms within the last 12 months and referral information | 3 months | No statistically significant differences in the TDI at the community or GP levels, or by factorial design for any tumour group. For LC: community intervention vs control: Median TDI 114.5 vs 114 days, Mean Difference=0.06, 95%CI 0.39-0.5, p=0.79; GP intervention vs control: 115 vs 125 days; Mean Difference=0.02, 95%CI 0.56-0.60, p=0.45 |
| Guldbrandt et al. (2014)  Denmark | To describe  the usage and outcome of a technological upgrade in a GP update format and implementing direct access to chest low dose  computed tomography (LDCT) from general practice for patients with respiratory symptoms | Cohort study nested in an RCT | n=133 GPs (64 participated in continuing medical education [CME] and 69 did not participate in CME)  60 general practices and Department of Radiology in a University Hospital | Amount of diagnostic workup needed  Cancer incidence  Use of fast-track referral option for suspected  LC  Stage at diagnosis | *IG:* Six times within a 3-month period, GPs were informed by letter about the intervention. Letters included information concerning the referral procedures and indications for the CT to let GPs substitute the radiograph with chest LDCT when ruling out LC  GPs invited to sit in 1 of 8 1-hour small-group-based CME meetings on the state-of-the-art knowledge on LC early detection  Algorithms for positive predictive values (PPV) in primary care used  GPs received information about CT, how to use them, and how to interpret the reports  *CG:* GPs did not participate in CME | Data obtained from GP referral notes on symptoms, known diseases, and smoking history  Danish Lung Cancer Registry used for information on subsequent LC diagnosis  Danish Deprivation Index used for information on deprivation rates in different GP clinics  Health Service Registry used to gather information  about GP list size and age/gender distribution of patients listed  with the GP  Indirect sex-age standardisation used to compare referral rates between CME-attending GPs and non-attending GPs | 19 months | 91 (68.4%) GPs used direct CTs  Referral rate to direct CT was significantly (61%) higher (95%CI 54-66%) among GPs working in a clinic with one or more CME-participating GPs  335 patients referred to LC fast-track. Of those, 33 (10%) had confirmed LC diagnosis. Of those, 8 (23.5%) had early-stage LC and 26 (76.5%) had advanced LC  Referral rate to LC fast-track was 0.13 (95%CI 0.09 to 0.19) for CME-participating GPs vs 0.14 (95%CI 0.09-0.20) for non-participating GPs (p=0.503)  PPV for LC diagnosis as a result of referral to a fast-track LC pathway was 13.3% (95%CI 8.7 to 19.1%) for CME-participating GPs and 6.1% (95%CI 3-11%) for non-participating GPs (p=0.027; i.e., 2.2 higher PPV) |
| Guldbrandt et al. (2015)  Denmark | To measure the effect of direct access to LDCT  from general practice in early LC detection on time to diagnosis and stage  at diagnosis | Cluster RCT | n=266 GPs (n=133 IG and n=133 CG)  119 General practices and Department of Radiology in a University Hospital | Primary care interval  Diagnostic interval  Stage at diagnosis | *IG:* Six times within a 3-month period, GPs were informed by letter about the intervention. Letters included information concerning the referral procedures and indications for the CT  GPs invited to sit in 1-hour small-group-based CME meetings to increase their awareness of LC  GPs received information about the CT, how to use them, and how to interpret the reports  If nodules (4-10 mm) could not be categorised as benign, GP referred patient to follow-up program (3, 6, or 12 months after first scan) as decided by the chest physicians. If CT revealed suspicion of LC, GP referred patients (fast track) to standard diagnostics  *CG:* Usual care | Danish Lung Cancer Registry and the Danish National Patient Registry used to identify LC cases  Danish Deprivation Index used to gather information about deprivation level in the different GP clinics’ population  Data on patient comorbidity obtained from GP Questionnaire  Data on identified LC patient’s socio-economic position collected from Statistics Denmark | 3, 6, or 12 months after the  first scan (according to the size and the characteristics of  the nodules) | No statistically significant difference in primary care interval between patients in IG (Median=14 days, inter quartile intervals [IQI]=4-53) and patients in CG (Median=18 days, IQI=5-69, prevalence ratio [PR]=0.99, 95%CI 0.65-1.54, p=0.455)  No statistically significant difference in diagnostic interval between patients in IG (Median=44 days, IQI=17 83) and patients in CG (Median=36 days, IQI=17-112, PR=0.8, 95%CI 0.5-1.27, p=0.299)  Primary care interval and diagnostic interval in IG statistically significantly shorter if the GP participated in CME (primary care interval Median=9 days [with CME] vs. 37 days [without CME], p=0.048; diagnostic interval Median=23 days [with CME] vs. 66 days [without CME], p=0.008)  Non-statistically significant higher risk of having a long diagnostic interval for patients in the CG (Risk difference=13.5%, 95%CI -11-37.9%, p=0.280). No statistically significant difference in risk for having a long primary care interval was observed using this approach (RD=1.1%, 95%CI 23.9 to 26.1%, p=0.929)  Non-statistically significant difference in stage of LC at diagnosis between CG and IG for all patients (p=0.586 for advanced [stage IV] LC and p=0.595 for localised [stage IA-IIIA] LC)  Non-statistically significant difference in stage of LC at diagnosis between CG and IG for patients whose GP was involved in the diagnosis (p=0.47 for advanced [stage IV] LC and p=0.658 for localised [stage IA-IIIA] LC) |
| Philips et al. (2021)  United States of America | To minimize diagnostic redundancy, streamline management decisions for indeterminate nodules, and expedite curative therapy for LC in patients at high-risk for treatment delay | Retrospective review of the Lung Cancer Strategist Programme (LCSP) | n=200 patients (n=100 LCSP patients and n=100 routine referral patients)  (n=78 LCSP and n= 41 routine referral patients managed via nodule surveillance program.  n= 22 LSCP and n=59 routine referral patients treated for an intrathoracic malignancy)  Thoracic surgery clinic | Timeliness of care delivery  Care efficacy  Patient care adherence  Stage at diagnosis  Disease Free Survival (DFS)  Overall Survival  (OS) | LCSP was led by a thoracic-trained advanced practice provider with training in lung nodule detection and treatment. The LCSP was designed to minimize diagnostic redundancy, streamline management decisions for indeterminate nodules, and expedite curative therapy for LC patients at high-risk for treatment delay | Following referral, a clinical strategist reviewed patients’ medical record, consulted with oncology specialists, developed an evaluation strategy for each patient, and ordered testing prior to any visit  Patients were first seen in clinic by their “Personalized Care Team” to review results, diagnosis and implement treatment  “Patients deemed high-risk for treatment delay, based on vulnerability criteria, presenting with a suspicious lung finding were accrued  prospectively via the LCSP versus routine surgical referral as the comparison group for this study” (p.2)  A retrospective review was performed of the first 100 patients managed via the LCSP vs routine referral | *Surgery:*  Median duration of follow up for LSCP patients (33 months); routine referral patients (31 months)  *Continued Surveillance:*  Median duration of follow up  for LSCP patients (27.5 months); routine referral patients (30months)  *Stereotactic body radiation therapy:* Median duration of follow up for LSCP patients  (21 months); RR patients (37 months) | Time from suspicious finding to initiation of workup significantly shorter in the LCSP vs routine referral (3 vs 28 days, p<0.001). Following referral, median time to workup also significantly shorter in the LCSP vs routine referral (1 vs 7 days, p<0.001)  Time from suspicious radiologic finding to definitive management plan=14.5 days in LCSP cohort vs 46.5 days in routine referral (p<0.001)  Referral to the LCSP moved patients into surveillance 1 month earlier relative to routine referral (12.5 vs 39 days, p<0.001). Following designation to nodule surveillance, 33 LCSP (42.3%) and 9 routine referral (21.9%) patients were discharged from care early in the process  As compared to routine referral, management via LCSP reduced the median number of hospital trips (4 vs 6, p<0.001), clinicians seen (1.5 vs 2, p=0.08), diagnostic studies obtained (4 vs 5, p=0.01), time from suspicious finding to LC diagnosis (30.5 vs 48, p=0.02) and treatment (40.5 vs 68.5, p=0.02)  Time to surgical resection significantly shorter in patients managed by LCSP vs routine referral (38 vs 69 days, p=0.05). Among patients with early-stage non-small cell LC treated with stereotactic body radiation therapy, the LCSP led to a substantial reduction in the time from suspicious finding to initiation of treatment in comparison to routine referral (62.5 vs 122.5 days, p=0.08).  No significant difference in stage at diagnosis for the 7 LCSP and 33 routine referral patients who underwent surgery for non-small cell LC. 6 of 7 LCSP patients (85.7%) had early-stage LC a median time from suspicious imaging to treatment of 37 days. In these 6 patients, DFS and OS were 100% (i.e., no recurrence or death) with a median duration of follow up of 33 months. In routine referral, 25 of 33 patients (75.7%) had early-stage disease with a median time from suspicious imaging to treatment of 68 days. In these 25 patients, there have been 6 recurrences (DFS=76%) and no deaths (OS=100) with a median duration of follow up of 35 months |
| Prades et al. (2011)  Spain | To reduce the time between well-founded suspicion of breast, colorectal and LC and the start of treatment | Mixed -methods study  of a Cancer Fast-track Programme (CFP) | n=56,020 individuals included in the CFP (quantitative)  n=83 HCPs (qualitative)  The Catalonian Health Service (private and publicly owned health facilities) | LC patients diagnosed through CFP route  Patients referred from GPs  Compliance with referral guidelines  LC detection rate  Mean time  between detection of suspected cancer and start of treatment  Distribution of the wait | CFP programme  aims to reduce the lag (time elapsed) between suspicion, diagnosis, and treatment of cancer, by designing circuits that would foster the rapid  coordination of the process circuit  Healthcare authorities issued organisational recommendations for effective implementation of these circuits, for  example, clinician responsible for disease, definition of maximum waiting times for diagnosis, study without hospitalisation where possible or coordination mechanisms in the event of referral to another hospital | Quantitative analysis of the CFP was performed using data generated by the hospitals based on seven FastTrack monitoring indicators for the period 2006-2009. All new cancer diagnoses were included but  cases of relapse were excluded | Not reported | Decrease in the proportion of LC patients diagnosed through the CFP route from 2006 (60.2% [95%CI 59.8-63.4]) to 2009 (53.2% [95%CI 51.5-54.9%])  Decrease in the proportion of LC patients referred by a GP from 2006 (60.6% [95%CI 59-62.3]) to 2009 (41.4% [95%CI 39.7-42.9%])  LC detection rate decreased from 49.9% (95%CI 48.2-51.6) in 2006 to 39.7% (95%CI 38.1-41.2%) in 2009  Mean time from detection of suspected LC in primary care to start of initial treatment increased from 30.8 days (2006) to 36.7 days (2009)  Increase in proportion of LC patients waiting over 45 days from the time of detection of suspected cancer to start of initial treatment (13.6% in 2006 vs 22.6% in 2009) Increase in proportion of LC cases waiting between 30-45 days (23.7% in 2006 to 26.1% in 2009)  Increase in compliance with referral guidelines from 70.8% in 2006 (95%CI 69.1-72.1) to 82.3% in 2009 (95%CI 81.1-83.5) |

**Abbreviations:** ALT=Alanine Aminotransferase; CFP=Cancer Fast-track Programme; CG=Control Group; CI=Confidence Interval; CME=Continuing Medical Education; DFS=Disease Free Survival; GP=General Practitioner; HCP=HCP; IG=Intervention Group; IQI=Inter Quartile Interval; IRR=Incidence Rate Ratio; LC=Lung Cancer; LCSP=Lung Cancer Strategist Programme; LDCT=Low Dose Computed Tomography; MDT=Multidisciplinary Team; OS=Overall Survival; PPV=Positive Predictive Value; PR=Prevalence Ratio; RCT=Randomised Controlled Trial; TDI=Total Diagnostic Interval
